# The Combined Association of Hearing Loss and Social Isolation on Dementia

**DOI:** 10.1093/geroni/igaf122.2926

**Published:** 2025-12-31

**Authors:** Xi Wang, Jason Smith, Thomas Cudjoe, Jennifer Schrack, Nicholas Reed, Jennifer Deal, Pablo Martinez-Amezcua

**Affiliations:** Johns Hopkins University Bloomberg School of Public Health, Baltimore, Maryland, United States; Johns Hopkins Bloomberg School of Public Health, Baltimore, Maryland, United States; Johns Hopkins School of Medicine, Baltimore, Maryland, United States; Johns Hopkins Bloomberg School of Public Health, Baltimore, Maryland, United States; New York University Grossman School of Medicine, New York, New York, United States; Johns Hopkins Bloomberg School of Public Health, Baltimore, Maryland, United States; Johns Hopkins Bloomberg School of Public Health, Baltimore, Maryland, United States

## Abstract

Hearing loss (HL) and social isolation (SI) are considered independent, modifiable risk factors for dementia. However, the modifying role of SI in the HL–dementia relationship remains unclear. We conducted a cross-sectional analysis of community-dwelling adults aged ≥65 in the U.S. from the nationally representative 2022 National Health and Aging Trends Study (NHATS) (n = 4,712). HL was defined as a better-ear pure tone average >25 decibels using a tablet-based auditory assessment. We measured binary SI using a four-domain typology (living arrangement, core discussion network, religious services attendance, and social participation). Dementia was evaluated using the NHATS dementia algorithm (reported dementia diagnosis, AD8, and neurocognitive tests). We used survey-weighted Poisson regressions to test if the additional presence of SI among older adults with hearing loss was associated with a higher risk of dementia. The prevalence of HL, SI, both combined and dementia, were 56.0%, 21.9%, 14.4%, and 9.2%, respectively. Compared to those without HL or SI, the prevalence ratio for dementia was 1.95 (95% CI: 1.38, 2.75) for HL alone, and 1.30 (95% CI: 0.75, 2.25) for SI alone, and 2.42 (95% CI: 1.66, 3.52) for those with both conditions. The super-additive interaction between HL and SI was not statistically significant (Synergy Index = 1.1, 95% CI: 0.4, 1.9). These findings suggest the presence of both HL and SI is associated with a higher prevalence of dementia compared to either factor alone. While no significant interaction was observed, HL management should consider coexisting isolation for dementia prevention.

